# Use of Oxford Nanopore MinION to generate full-length sequences of the *Blastocystis* small subunit (*SSU*) rRNA gene

**DOI:** 10.1186/s13071-020-04484-6

**Published:** 2020-11-25

**Authors:** Jenny G. Maloney, Aleksey Molokin, Monica Santin

**Affiliations:** Environmental Microbial and Food Safety Laboratory, Agricultural Research Service, United States Department of Agriculture, Beltsville, MD USA

**Keywords:** *Blastocystis*, Long-read sequencing, MinION, Ribosomal RNA, Subtypes

## Abstract

**Background:**

*Blastocystis* sp. is one of the most common enteric parasites of humans and animals worldwide. It is well recognized that this ubiquitous protist displays a remarkable degree of genetic diversity in the *SSU* rRNA gene, which is currently the main gene used for defining *Blastocystis* subtypes. Yet, full-length reference sequences of this gene are available for only 16 subtypes of *Blastocystis* in part because of the technical difficulties associated with obtaining these sequences from complex samples.

**Methods:**

We have developed a method using Oxford Nanopore MinION long-read sequencing and universal eukaryotic primers to produce full-length (> 1800 bp) *SSU* rRNA gene sequences for *Blastocystis*. Seven *Blastocystis* specimens representing five subtypes (ST1, ST4, ST10, ST11, and ST14) obtained both from cultures and feces were used for validation.

**Results:**

We demonstrate that this method can be used to produce highly accurate full-length sequences from both cultured and fecal DNA isolates. Full-length sequences were successfully obtained from all five subtypes including ST11 for which no full-length reference sequence currently exists and for an isolate that contained mixed ST10/ST14.

**Conclusions:**

The suitability of the use of MinION long-read sequencing technology to successfully generate full-length *Blastocystis SSU* rRNA gene sequences was demonstrated. The ability to produce full-length *SSU* rRNA gene sequences is key in understanding the role of genetic diversity in important aspects of *Blastocystis* biology such as transmission, host specificity, and pathogenicity.
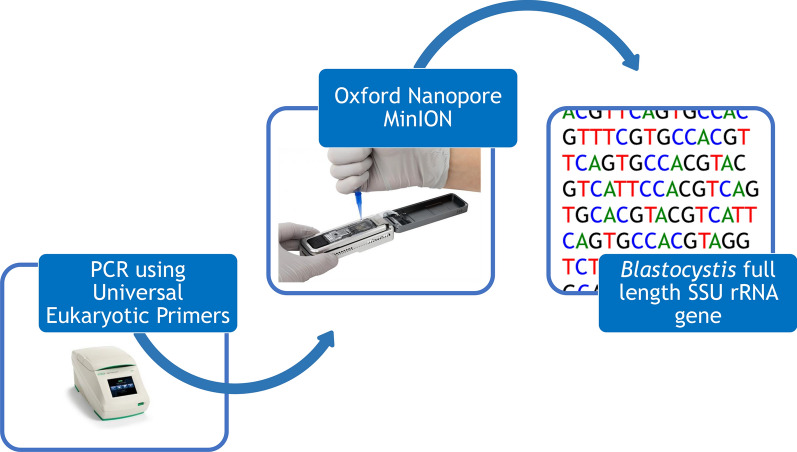

## Background

*Blastocystis* sp. is a common enteric protist parasite of humans and animals [1, 2]. It has a global distribution and is one of the most common human intestinal parasites in both developed and developing countries [3, 4]. Infection with *Blastocystis* in humans has been linked to gastrointestinal illnesses and/or urticaria [5, 6]. However, the pathogenicity of *Blastocystis* remains a topic of some controversy as asymptomatic infection is also commonly reported [7, 8]. *Blastocystis* transmission occurs via the fecal-oral route. Infections can be acquired through direct or indirect transmission (waterborne and foodborne) [9–11]. Yet many aspects related to the transmission and zoonotic potential of *Blastocystis* remain to be elucidated.

*Blastocystis* sp. is currently classified as a stramenopile based first on a small subunit (*SSU*) of the ribosomal RNA (rRNA) gene phylogeny and later supported by other genes [12, 13]. Although *Blastocystis* specimens isolated from humans and animals are morphologically indistinguishable, the application of molecular methods has shown significant genetic diversity among specimens from both humans and animals. Within the genus *Blastocystis*, 28 genetic groups named as subtypes (STs) have been proposed, based on polymorphism in the *SSU* rRNA gene [14, 15]. Of the 28 proposed subtypes, 22 subtypes (ST1–ST17, ST21, ST23–ST26) have been acknowledged as legitimate subtypes meeting the criteria of having *SSU* rDNA sequences that differ by 4% or more [14, 16]. These subtypes have been named using a numbering system that is currently sequential and based on publication date. Of the ten subtypes reported in humans, nine (ST1-ST8 and ST12) have also been reported in animal samples indicating that this parasite may have a zoonotic transmission cycle.

*Blastocystis* sp. genetic diversity is quite remarkable considering that novel subtype designations currently require a divergence of > 4% in the sequence identity from any named subtype [14]. As interest in the field of *Blastocystis* molecular epidemiology grows and new hosts and geographic regions are sampled, it seems likely that more novel subtypes will continue to emerge. While not universally adopted, it has been suggested that new subtype designations only be assigned if an almost full-length *SSU* rRNA gene sequence (> 80%) has been produced and demonstrated through comparison to other full-length sequences to meet the 4% divergence threshold [14]. While this requirement may be easily achieved for some *Blastocystis* subtypes that can be obtained in pure culture, most *Blastocystis* subtypes are currently not available in culture. And like other protist parasites, the culture conditions needed for one subtype may not work for others [17]. Thus, a culture-free method to obtain full-length reference sequences directly from fecal specimens is needed. Currently, near complete *SSU* rRNA gene sequences are only available for ST1–ST10 and ST12–ST17, and there is a clear need to obtain full-length sequences for the other proposed subtypes to be validated. This information can be used to determine the validity of the proposed subtypes using the 4% divergence across 80% of the gene naming system as well as to conduct phylogenetic analysis to established clade structure when the full length, and not a partial region, of the *SSU* rRNA gene is used for all *Blastocystis* subtypes currently proposed.

MinION is the first commercial nanopore sequencer developed by Oxford Nanopore Technologies (ONT). It can be defined as a third-generation sequencing platform considering its single-molecule sequencing ability, but its technical principles and properties are very different compared with the previous platforms [18]. MinION is a palm-sized device that drives individual DNA/RNA molecules through a nanopore; only a single strand nucleic acid can pass through the pore. Because the electrical properties of the bases A, T, G, and C are different, electrical signals with base specificity can be detected by MinION and sequence information can thus be collected continuously using the MinKNOW software. MinION is capable of generating reads as long as 882 kb, which improves the scaffolding of prokaryotic and eukaryotic genomes [19]. The ability to generate long reads also has applications outside of genome studies such as sequencing of full-length genes used in taxonomic and epidemiological studies [20–23].

In the present study, we developed a method for generating full-length *Blastocystis SSU* rRNA gene sequences using the MinION long-read sequencing technology from amplicons obtained using universal eukaryotic *SSU* rRNA gene primers. The method was validated using *Blastocystis* DNA obtained from both cultured and fecal samples. Moreover, we compared the data with Illumina MiSeq sequencing results.

## Methods

### Source of *Blastocystis* isolates

Seven DNA samples containing *Blastocystis* from both cultured and fecal isolates were used in this study (Table 1). Cultured isolates were obtained from ATCC, and fecal isolates were selected from an archive of *Blastocystis*-positive DNA samples. All isolates were typed using a PCR that amplifies an approximately 500 bp region of the *SSU* rRNA gene suitable for *Blastocystis* subtype differentiation using Sanger and next-generation sequencing using previously reported protocols [24, 25] (Table 1). Illumina Miseq library preparation and bioinformatic analysis were performed as previously described [25].

**Table 1:** Information of *Blastocystis* specimens used in this study including host, geographic origin, and subtype

Specimen ID	Host	Location	*Blastocystis* subtype
1	Human^a^	USA	ST1
2	Human^b^	USA	ST4
3	Human^c^	Spain	ST4
4	Elephant	USA	ST11
5	Cattle^d^	USA	ST10
6	Cattle	USA	ST10/ST14
7	Cattle	USA	ST14

^a^Isolate acquired from ATCC (Blastocystis ATCC 50177^™^)

^b^Isolate acquired from ATCC (Blastocystis ATCC 50608^™^)

^c^Isolate H-1 reported in Santin et al. [24]

^d^Isolate C-3073 reported in Santin et al. [24]

### PCR amplification of the full-length *SSU* rRNA gene

The approximately 1800 base pair *SSU* rRNA gene was amplified by PCR using the primers Af (5′-AAC CTG GTT GAT CCT GCC AGT AGT C-3′) and Br (5′-TGA TCC TTC TGC AGG TTC ACC TAC G-3′), which amplify the *SSU* rRNA gene of most eukaryotic organisms [26, 27]. Amplification was performed as previously described with the exception that the high-fidelity proofreading polymerase contained in KAPA HiFi HotStart ReadyMix (KAPABioSystems, Cape Town, South Africa) was used. The reaction used 1 µM forward and reverse primers and 12.5 µl of KAPA HiFi HotStart ReadyMix in a 25 µl reaction volume. Following amplification, PCR products were visualized using a QIAxcel (Qiagen, Valencia, CA, USA) and quantified using a Qubit fluorometer (ThermoFisher Scientific, Waltham, MA, USA).

### Performing platform quality control

Prior to sequencing, a hardware check was performed via the MinKNOW software using the Configuration Test Cell to ensure successful communication between MinION and software. A flow cell check was performed prior to every sequencing run to determine that a sufficient number of active pores was available in each flow cell. To maximize MinION yield and reduce the time during which nanopores are idle, amplicons were quantified and diluted to make sure 100–200 fmol of DNA was used as input into library prep as recommended by ONTs instructions for amplicon sequencing on the MinION.

### Nanopore library construction and sequencing

Nanopore sequencing libraries were prepared from each amplicon using Oxford Nanopore Technologies (ONT) SQK-LSK109 1D Ligation Sequencing Kit (ONT, Oxford, UK) following the manufacturer’s protocol for 1D amplicon/cDNA by Ligation (version: ACDE_9064_v109_revG_23May2018). Briefly, the library preparation involves repairing amplicon ends, preparing the ends for nanopore adapters, and attaching the sequencing adapters. Based on protocol recommendations, 100–200 fmol of amplicon was used as input for library construction, and 12 µl of each library was loaded onto the flow cell for sequencing. The nanopore sequencing libraries were individually run on R9.4 flow cells (FLO-MIN106) using an ONT MinION Mk1B and basecalled using the MinIT (ONT-MinIT Release 19.06.08). ATCC strains were sequenced for 1 h to approximately 250,000 reads. All other samples were sequenced to approximately 500,000 reads or until the flow cell’s output plateaued (Table 2).

**Table 2:** Bioinformatic analysis data for each step in processing of MinION sequences obtained from the specimens used in this study

Specimen ID	1	2	3	4	5	6	7
Total MinION reads	260,471	272,100	574,327	562,189	509,070	505,177	335,913
Total Mbases	458.1	510	887.2	547.9	657.2	474.4	259.5
Reads > Q7	236,039	247,399	515,724	529,182	431,051	456,117	296,619
Mbases > Q7	428.1	479	815.5	524.3	574.4	434.6	233
Reads with length 1000–2100 nt	93,828	89,141	131,127	105,440	109,609	87,019	55,970
Reads after canu correction	79,682	61,484	77,149	69,586	57,435	50,107	28,966
Reads after canu trimming	71,895	55,643	46,519	44,990	29,693	27,873	19,805
Strand (+) reads with both forward and reverse primers	15,730	9,573	7,310	8,734	4,620	3,430	1,376
Strand (−) reads with both forward and reverse primers	14,242	9,407	5,646	6,427	3,816	2,616	986
Strand (+) and (−) reads combined	29,972	18,980	12,956	15,161	8,436	6,046	2,362
Reads that aligned to a *Blastocystis* reference with > 90% identity and coverage	26,314	17,960	4,626	2,581	4,309	1,077	287
No. clusters after clustering reads at 98% identity	34	4,556	265	256	682	152	47
No. clusters with an abundance > 5	18	25	12	7	11	7	3
Total abundance of all clusters that had at least 5 reads	26,116	10,674	4,353	2,158	3,585	725	30
No. clusters that aligned to expected *Blastocystis* reference after racon polishing and re-clustering	1	1	1	2	1	2	1
No. clusters after nanopolishing and re-clustering	1	1	1	2	1	2	1

### Bioinformatic analysis

Basecalling was performed using ONT Guppy v3.0.4 aboard the MinIT data processing unit (ONT-MinIT-Release 19.06.8) using a minimum quality score of 7 for filtering low-quality reads. All FASTQ files within each sample were concatenated into a single file and filtered to only include reads between 1000 and 2100 nucleotides in length. Reads were then corrected and trimmed using Canu v1.9 [28] with the following parameters: *-correct*, *genomeSize* = 1.7 k, *minOverlapLength* = 1000, *corOutCoverage* = 1000000; *-trim trimReadsCoverage* = 20. Next, reads containing intact forward and reverse primer sequences were extracted using *bbduk.sh* (*k* = 18, *restrictleft/right* = 500, *rcomp* = *f*, *mm* = *f*, edist = 2) via BBTools v38.55 [29], and primer sequences were queried to establish plus and minus strand reads separately. Minus strand reads were then reverse complemented and combined with plus strand reads into a single FASTA file. To filter out off-target reads, a *Blastocystis* reference database was downloaded from NCBI using the following criteria: “*blastocystis [ORGN] AND 0:6000 [SLEN] AND biomol_genomic[PROP]*.” The FASTA file containing the reference sequences was indexed using VSEARCH v2.14.1 [30] with *vsearch --makeudb_usearch* command. Read filtering was then performed using the *vsearch --usearch_global* command with the following parameters: *--id 0.9 --query_cov 0.9*. Next, consensus sequences were generated by clustering reads using the *vsearch --cluster_fast* command with a 98% identity threshold. Consensus sequences were checked for chimeras using the *vsearch --uchime_denovo* command and then filtered using a minimum abundance threshold of 5. Sequences were polished using Racon v1.4.11 [31]. The alignment file needed for polishing was generated using Minimap2 v2.17-r941 [32] (*-ax asm5 --secondary* = *no*) by mapping the VSEARCH filtered reads to the chimera-free sequences. Polishing was then performed using default Racon parameters. Polished sequences were clustered again at a 98% identity threshold and prepared for another round of improvement with Nanopolish v0.11.1 [33] to leverage signal-level FAST5 data. The reads used for this step were Canu-corrected, trimmed reads that were down-sampled using *bbnorm.sh* to a target coverage of 500. Down-sampled reads were mapped to the Racon-polished, re-clustered consensus sequences using Minimap2 (*-ax asm5 --secondary* = *no*), and the alignment file was sorted and indexed using Samtools v1.9 [34]. Polishing was executed using the *nanopolish variants --consensus* command with the parameters *--min-flanking-sequence* = 10, *--fix-homopolymers*, and *--max-haplotypes* = 1000000. The *nanopolish vcf2fasta* command was then used to apply the improvements from the previous step to the Racon-polished, re-clustered consensus sequences. Nanopolished sequences were re-clustered once more at a 98% identity threshold to obtain final consensus sequences. Subtypes were assigned based on the best match to a reference in the GenBank database using BLAST. The nucleotide sequences obtained in this study have been deposited in GenBank under the accession numbers MT898451–MT898459.

For comparison purposes, for each same sample, full-length sequences and partial sequences obtained with MinION and MiSeq, respectively, were aligned using ClustalW in MegAlign 15 (DNASTAR Lasergene 15, Madison, WI, USA), and pairwise distances between consensus sequences were calculated.

## Results

Nanopore sequencing of seven *Blastocystis* isolates representing five subtypes (ST1, ST4, ST10, ST11, and ST14) was performed to test the use of this method for producing full-length *SSU* rRNA gene reference sequences from complex samples such as feces. Full-length sequences were successfully obtained from all five subtypes including ST11, for which no full-length reference sequence currently exists, and for an isolate that contained mixed ST10/ST14 (Table 1). Samples were individually sequenced to a depth of between 250,000 and 575,000 reads (Table 2). A stringent filtering procedure, which included the removal of sequences that did not include both the forward and reverse primer sequences, reduced the total reads available for consensus generation to between 2000 and 26,000 reads per sample (Table 2).

To obtain high-quality consensus sequences, Racon-polished consensus sequences were further refined using Nanopolish. This step improved consensus quality by filling gaps in homopolymer regions, correcting substitution errors, and removing artifactual sequences from the ends of reads (Additional file 1: Figure S1). In an alignment between the MinION generated consensus sequence for the ST1 isolate from ATCC 50177 (#1) and a previously published Sanger sequence from this same isolate (GenBank accession no. U51151), only one disagreement in sequence identity was present outside the primer region, a missing base in a homopolymer region at approximately 669 bp (Additional file 1: Figure S1). In fact, sequence identity between the best match from GenBank and the MinION generated consensus sequence for all the isolates sequenced in this study was high, ranging from 98.6 to 100% (Table 3). Likewise, sequence identity between the Illumina generated sequences for a fragment of the *SSU* rRNA gene and same region of the MinION consensus sequence was very high ranging from 99.8 to 100%. Thus, high-quality full-length *Blastocystis SSU* rRNA reference sequences can be generated using this method.

**Table 3:** Comparison of full-length *Blastocystis* SSU rRNA gene sequences generated in this study by MinION sequencing to Illumina MiSeq sequences from the same sample and closest full-length match available on GenBank

Specimen ID	*Blastocystis* subtype	Length of sequence generated by MinION in this study (GenBank accession number)	Similarity to Illumina MiSeq sequence^a^ (%)	Similarity to closest match available in GenBank (sequence length, accession number)
1	ST1	1766 bp (MT898451)	100	99.8 (1770 bp, U51151)
2	ST4	1772 bp (MT898452)	100	100 (1730 bp, AY590114)
3	ST4	1773 bp (MT898453)	100	99.9 (1730 bp, AY590114)
4	ST11	1762 bp (MT898454)	100	99.9 (989 bp, GU256903)
		1763 bp (MT898455)	100	98.6 (989 bp, GU256929)
5	ST10	1770 bp (MT898456)	99.8	99.5 (1728 bp, KC148207)
6	ST10	1770 bp (MT898457)	99.8	99.5 (1728 bp, KC148207)
	ST14	1771 bp (MT898458)	99.8	99.6 (1772 bp, KC148205)
7	ST14	1771 bp (MT898459)	99.8	99.7 (1772 bp, KC148205)

^a^Illumina MiSeq sequence corresponds to the approximately 480 bp region of the SSU rRNA gene amplified using the primers reported in [11] using methods reported in [12]

Sequence coverage varied between samples with cultured isolates having more reads retained for consensus generation than fecal isolates (Table 2). However, even consensus sequences generated from lower sequence coverage such as the ST14 from sample #6 (18× coverage) and ST14 from sample #7 (30× coverage) compared favorably in accuracy to both Illumina and GenBank sequences (Table 3). As such, reference sequences from samples with proportionally low *Blastocystis* amplicon can be produced with this method.

Multiple subtypes or subtype variants were detected in two samples by MinION sequencing. Sample #4 contained two variants of ST11, which were supported by Illumina sequence data as well as by having different best matches in GenBank. Sample #6 contained ST10 and ST14 with ST10 being the majority of sequence present. This was in agreement with Illumina sequence data obtained for this sample where ST10 represented the majority of the *Blastocystis* sequence. Thus, even mixtures of *Blastocystis* subtypes can be differentiated with this method.

## Discussion

Full-length *SSU* rRNA gene sequences of *Blastocystis* provide useful reference sequences for both subtype identification and the production of phylogenies, which can attempt to recreate the relationships between subtypes of this common parasite of humans and animals. It has also been recommended that new subtype designations only be assigned if a full-length *SSU* rRNA gene sequence has been produced (> 80% of the approximately 1800 bp *SSU* rRNA full-length) and demonstrated through comparison to other full-length sequences to meet a minimum 4% divergence threshold from any named subtype [14, 35]. However, to achieve full-length sequences using current methodology requires using multiple primer sets to sequence multiple PCR products, which are sequenced using Sanger sequencing and then pieced together to produce the full-length or almost full-length *SSU* rRNA gene [36]. The use of multiple primer sets and Sanger sequencing to produce full-length *SSU* rRNA gene sequences is not only laborious but may be complicated for subtypes that are not available in culture. For example, issues with primer affinity or mixed subtypes present in DNA extracted directly from a fecal specimen could make accurate subtype identification impossible using the Sanger sequencing method. These issues may in part explain why full-length sequences do not exist for all of the named subtypes of *Blastocystis*.

First-generation sequencing (Sanger dideoxy chain-termination method) has long served as the standard method for production of *Blastocystis* sp. reference sequences and is both widely available and relatively cheap to perform. However, it does not have the ability to discern mixed infections nor can it produce full-length sequences as the maximum sequence length is currently around 1000 nucleotides. Second-generation sequencers such as Roche 454 pyrosequencing, Illumina, Solexa, and ABI SOLiD systems are capable of massively parallel sequencing, which can resolve complex mixtures of amplicon such as those found when mixtures of multiple *Blastocystis* subtypes are present in the same host. These systems do not produce long reads, however, and like Sanger sequencing the production of full-length gene sequences requires combining amplicon sequences from multiple PCR reactions to achieve a full-length sequence. Third-generation sequencing platforms like the ONT MinION offer three major advantages over first- and second-generation sequencing methods: (i) increase in read length from tens of bases to tens of thousands of bases per read; (ii) reduction of sequencing time from days to hours (or to minutes for real-time applications); (iii) reduction or elimination of sequencing biases introduced by PCR amplification [37–39]. The MinION sequencer, due to its small size and low equipment cost, is attracting considerable interest in the genomics community. However, in its early iterations the platform suffered from high error rates that resulted in individual raw read accuracies ranging from 65 to 88% and a limited output ranging from 0.1 to 2 Gb of raw sequence data [19, 40–43]. Since its inception, these MinION shortcomings have fueled significant efforts to improve the nanopore instrumentation, pore chemistry, and software used for basecalling and post-assembly/consensus polishing. With more recent advances in MinION technology yields reported within the past 2 years range from 5 to 10 Gb while the most recent output numbers directly from ONT are 15–30 Gb depending on the sample type and library prep method. Recent improvements in basecalling and post-processing software have led to individual read accuracies in the range of 85–95% and consensus accuracies > 99% [19, 44–46].

As an alternative to traditional Sanger sequencing, long-read sequencing platforms provide a useful tool for addressing issues related to the production of *Blastocystis* sp. full-length reference sequences. They can generate individual reads that are several kilobases in length, removing the need for the use of multiple primers and PCRs. Furthermore, as ONT sequencing platforms such as the MinION also produce thousands of reads from a single sample, it provides the sequencing depth needed to discern mixtures of sequences which would allow for the detection of multiple subtypes within a single sample, removing the need for pure cultures of parasite to obtain full-length reference sequences. In this study, we have developed a sequencing protocol and analysis pipeline for producing *Blastocystis* full-length reference sequences using the Oxford Nanopore MinION. We tested this protocol using DNA extracted from both cultured and fecal isolates of *Blastocystis* and found we could successfully produce full-length *SSU* rRNA sequences from both sample types (Table 3).

To determine the reliability of reference sequences generated using the MinION and data processing methods described in this study, a cultured *Blastocystis* ST1 isolate for which a full-length Sanger sequence is published was included in this study and sequenced using the MinION (sample #1). Sample #1 (ATCC 50177) was sequenced to a depth of 270,000 sequences, which after stringent filtering produced a reference sequence of 1766 base pairs in length composed of 5400 reads. After polishing, the sequence obtained in this study shares 99.8% sequence identity with the Sanger sequence of the same isolate on GenBank (U51151) (Table 3). In fact, outside of the primer regions there is only one difference between the polished consensus sequence produced by MinION sequencing and the reference Sanger sequence. This difference is a missing base in a homopolymer region at approximately 669 base pairs in the MinION sequence (Additional file 1: Figure S1). It is known that Nanopore sequencing has trouble with homopolymer sequences [33, 47–49]. These issues can be observed in the unpolished sequence in Additional file 1: Figure S1. However, the polishing steps employed here were able to correct all but one of the errors present because of homopolymer stretches. Furthermore, the sequencing of the other ATCC isolate 50608 also yielded full-length *SSU* rRNA sequences with high sequence identity to GenBank reference sequences of the same subtype (Table 3). Together these results support Nanopore sequencing as a method for producing highly accurate reference sequences for *Blastocystis*.

Sequencing of five fecal isolates of *Blastocystis* was performed to assess the suitability of this method for producing full-length reference sequences from complex sample types. Full-length *SSU* rRNA sequences were successfully produced for ST4, ST10, ST11, and ST14. For sample #4 and #6, two sequence variants of ST11 and a mixture of ST10 and ST14, respectively were detected using this method (Tables 1 and 2). These results were confirmed by Illumina MiSeq amplicon sequencing of a fragment of the *SSU* rRNA gene of these samples, indicating that the long-read sequencing method described in this study can produce full-length reference sequences from complex sample types and can successfully detect mixed subtype infections and intra-subtype variability within a sample.

This study is the first to report a full-length reference sequence for ST11. To date, only partial reference sequences of this subtype have been reported, the longest of which is approximately 1000 base pairs in length [50]. We have produced two reference sequences for ST11 which were present in the same sample from an elephant. Both sequences are over 1760 base pairs in length, have different best matches in the GenBank database, and are supported with data from Illumina MiSeq sequences of a fragment of the same gene (Table 3). The need for a full-length ST11 reference sequence has been noted previously as currently the 600 bp region from the 5′ region of the gene which is commonly used for subtyping *Blastocystis* isolates has never been sequenced for ST11. A BLAST search of the first 600 base pairs of the full-length reference produced in this study returned a sequence match of 99% percent identity from an Asian elephant from a zoo in Bangladesh [51]. The nucleotide sequence from this Asian elephant displays in GenBank (MN338089) as an unpublished study with nucleotide sequence submitted in August of 2019. The submitters of the sequence give it a designation of ST23 in GenBank, which is problematic for two reasons. First, this sequence is ST11 (as indicated in their publication) [51]; second, the designation ST23 is already in use [52]. Thus, the reference sequences for ST11 produced in this study can aid in preventing the misassignment of ST11 sequences in the future.

Nanopore sequencing to produce full-length *SSU* rRNA sequences of *Blastocystis*, while a useful and novel tool, does present some challenges. For example, off-target amplification products were highly represented in the sequences even in the cultured *Blastocystis* isolates (Table 2). Even after stringent size and quality filtering, the number of off-target clusters produced from the sequenced isolates in this study outnumbered the target sequences in almost every sample. However, the majority of these sequences were of bacterial origin (data not shown) and can be easily removed from the final sequence pool. Thus, off-target amplification should not affect consensus calling. However, the predominance of these sequences in the sequencing pool means that the sequencing depth of a sample may need to be quite high to successfully capture the target sequence from complex sample. Furthermore, this issue may be amplified in complex samples where off-target amplification masks the presence of *Blastocystis* in samples where the parasite of interest is present in low abundance.

## Conclusions

The ability to successfully generate full-length *Blastocystis SSU* rRNA gene sequences using MinION long-read sequencing technology from *Blastocystis* DNA extracted from culture and fecal samples was demonstrated. Additionally, the first full-length reference sequence for ST11 was produced. This technique can be used to produce full-length reference sequences for novel subtypes and isolates containing mixed subtypes. This tool will be useful to obtain the near-complete *SSU* rRNA sequences recommended for naming novel subtypes to avoid the designation of invalid subtypes that will create confusion undermining subtype terminology. In addition, more robust phylogenetic analyses will be possible when near-complete *SSU* rRNA sequences are available for all subtypes. Lastly, longer *SSU* rRNA sequences may be useful in improving our understanding of the sources, transmission, pathogenicity, and host specificity of this genetically diverse parasite.

## Supplementary information

**Additional file 1: Figure S1.** Alignment of sample # 1 (ATCC 50177) generated in this study using Oxford Nanopore MinION unpolished and polish and reference sample U51151 generated using Sanger sequencing.

## Data Availability

The nucleotide sequences obtained in this study have been deposited in GenBank under the accession numbers MT898451–MT898459.

## Abbreviations

PCR: Polymerase chain reaction
ONT: Oxford Nanopore Technologies
*SSU* rRNA: Small subunit of the ribosomal RNA
ST: Subtype
