# Supplementary material for: Use of Oxford Nanopore MinION to generate full-length sequences of the Blastocystis small subunit (SSU) rRNA gene
Source: Parasit Vectors. 2020 Nov 25;13:595. doi: 10.1186/s13071-020-04484-6 (PMC7687777; doi:10.1186/s13071-020-04484-6)
Supplement: Supplementary file 1 — Additional file 1: Figure S1. Alignment of sample # 1 (ATCC 50177) generated in this study using Oxford Nanopore MinION unpolished and polish and reference sample U51151 generated using Sanger sequencing. [file 13071_2020_4484_MOESM1_ESM.pdf]

```
U51151.1    GCTTATCTGGTTGATCCTGCCAGTAGTCATACGCTCGTCTCAAAGATTAAGCCATGCATG
polished    ---AACCTGGTTGATCCTGCCAGTAGTCATACGCTCGTCTCAAAGATTAAGCCATGCATG
unpolished  ---AACCTGGTTGATCCTGCCAGTAGTCATACGCTCGTCTCAAAGATTAAGCCATGCATG
            1........10........20........30........40........50.........

U51151.1    TGTAAGTGTAAATATACTAGTTTGGAACTGCGAATGGCTCATTATATCAGTTATAGTTTA
polished    TGTAAGTGTAAATATACTAGTTTGGAACTGCGAATGGCTCATTATATCAGTTATAGTTTA
unpolished  TGTAAGTGTAAATATACTAGTTTGGAACTGCGAATGGCTCATTATATCAGTTATAGTTTA
            61.......70........80........90........100.......110........

U51151.1    TTTGGTGAAGTGTACTACTTGGATAACCGTAGTAATTCTAGGGCTAATACATGAGAAAGT
polished    TTTGGTGAAGTGTACTACTTGGATAACCGTAGTAATTCTAGGGCTAATACATGAGAAAGT
unpolished  TTTGGTGAAGTGTACTACTTGGATAACCGTAGTAATTCTAGGGCTAATACATGAGAAAGT
            121......130.......140.......150.......160.......170........

U51151.1    CCTCTGGTGAGGTGTGTTTATTAGAATGAAAACCATATGCTTCGGCATGATAGTGAGTAA
polished    CCTCTGGTGAGGTGTGTTTATTAGAATGAAAACCATATGCTTCGGCATGATAGTGAGTAA
unpolished  CCTCTGGTGAGGTGTGTTTATTAGAATGAAAACCATATGCTTCGGCATGATAGTGAGTAA
            181......190.......200.......210.......220.......230........

U51151.1    TAGTAACCTATCGTATCGCATGCTTAATGTAGCGATGAGTCTTTCAAGTTTCTGCCCTAT
polished    TAGTAACCTATCGTATCGCATGCTTAATGTAGCGATGAGTCTTTCAAGTTTCTGCCCTAT
unpolished  TAGTAACCTATCGTATCGCATGCTTAATGTAGCGATGAGTCTTTCAAGTTTCTGCCCTAT
            241......250.......260.......270.......280.......290........

U51151.1    CAGCTTTCGATGGTAGTATATGGGCCTACCATGGCAGTAACGGGTAACGAAGAATTTGGG
polished    CAGCTTTCGATGGTAGTATATGGGCCTACCATGGCAGTAACGGGTAACGAAGAATTTGGG
unpolished  CAGCTTTCGATGGTAGTATATGGGCCTACCATGGCAGTAACGGGTAACGAAGAATTTGGG
            301......310.......320.......330.......340.......350........

U51151.1    TTCGATTTCGGAGAGGGAGCCTGAGAGATGGCTACCACATCCAAGGAAGGCAGCAGGCGC
polished    TTCGATTTCGGAGAGGGAGCCTGAGAGATGGCTACCACATCCAAGGAAGGCAGCAGGCGC
unpolished  TTCGATTTCGGAGA-GGAGCCTGAGAGATGGCTACCACATCCAAGGAAGGCAGCAGGCGC
            361......370.......380.......390.......400.......410........

U51151.1    GTAAATTACCCAATCCTGACACAGGGAGGTAGTGACAATAAATCACAATGCGGGACTATC
polished    GTAAATTACCCAATCCTGACACAGGGAGGTAGTGACAATAAATCACAATGCGGGACTATC
unpolished  GTAAATTACCCAATCCTGACACAGGGAGGTAGTGACAATAAATCACAATGCGGGACTATC
            421......430.......440.......450.......460.......470........

U51151.1    AGTCTTGCAATTGGATTGAGAACAATGTACAACTCTTATCGATAAGCCATTGGAGGGCAA
polished    AGTCTTGCAATTGGATTGAGAACAATGTACAACTCTTATCGATAAGCCATTGGAGGGCAA
unpolished  AGTCTTGCAATTGGATTGAGAACAATGTACAACTCTTATCGATAAGCCATTGGAGGGCAA
            481......490.......500.......510.......520.......530........

U51151.1    GTCTGGTGCCAGCAGCCGCGGTAATTCCAGCTCCAATAGCGTATATTAACGTTGTTGCAG
polished    GTCTGGTGCCAGCAGCCGCGGTAATTCCAGCTCCAATAGCGTATATTAACGTTGTTGCAG
unpolished  GTCTGGTGCCAGCAGCCGCGGTAATTCCAGCTCCAATAGCGTATATTAACGTTGTTGCAG
            541......550.......560.......570.......580.......590........

U51151.1    TTAAAAAGCTCGTAGTTGAAGTGTGGGTGATCGCTGTTGTGAGACTTCGGTCTCTCGACA
polished    TTAAAAAGCTCGTAGTTGAAGTGTGGGTGATCGCTGTTGTGAGACTTCGGTCTCTCGACA
unpolished  TTAAAAAGCTCGTAGTTGAAGTGTGGGTGATCGCTGTTGTGAGACTTCGGTCTCTCGATA
            601......610.......620.......630.......640.......650........

U51151.1    GTAAGTCACCCCCTTCCAGTATCCAGTAGTGGGTATTCAGTTACTTACTACTGTGTGTTG
polished    GTAAGTCA-CCCCTTCCAGTATCCAGTAGTGGGTATTCAGTTACTTACTACTGTGTGTTG
unpolished  GTAAGTCA-CCCCTTCCAGTATCCAGTAGTGGGTATTCAGTTACTTACTATTGTGTGTTG
            661......670.......680.......690.......700.......710........

U51151.1    GTCCTTTACTGTGAGAAAATTAGAGTGTTCAAAGCAGGCGTTTGCTTGAATAGATTAGCA
polished    GTCCTTTACTGTGAGAAAATTAGAGTGTTCAAAGCAGGCGTTTGCTTGAATAGATTAGCA
unpolished  GTCGTTTACTGTGAGAAAATTAGAGTGTTCAAAGCAGGCGTTTGCTTGAATAGATTAGCA
            721......730.......740.......750.......760.......770........

U51151.1    TGGAATAATAATTGAAGGCTTTCGTGTTCGATTTGATTGGTTTGTTCATGGAAGCAAGGT
polished    TGGAATAATAATTGAAGGCTTTCGTGTTCGATTTGATTGGTTTGTTCATGGAAGCAAGGT
unpolished  TGGAATAATAATTGAAGGCTTTCGTGTTCGATTTGATTGGTTTGTTCATGGAAGCAAGGT
            781......790.......800.......810.......820.......830........
```

```
U51151.1    TAAAAGGAACAGTTGGGGGTATTCATATTCACTAGTTAGAGGTGAAATTCTCGGATTTAT
polished    TAAAAGGAACAGTTGGGGGTATTCATATTCACTAGTTAGAGGTGAAATTCTCGGATTTAT
unpolished  TAAAAGGAACAGTT-GGGGTATTCATATTCACTAGTTAGAGGTGAAATTCTCGGATTTAT
            841......850.......860.......870.......880.......890........

U51151.1    GGAAGATGAACAAGTGCGAAAGCATTTACCAAGGATGTTTTCATTAATCAAGAACGAAAG
polished    GGAAGATGAACAAGTGCGAAAGCATTTACCAAGGATGTTTTCATTAATCAAGAACGAAAG
unpolished  GGAAGATGAACAAGTGCGAAAGCATTTACCAAGGATGTTTTCATTAATCAAGAACGAAAG
            901......910.......920.......930.......940.......950........

U51151.1    CTAGGGGATCGAAGAGGATTAGATACCCTCGTAGTCTTAGCTATAAACGATACCGACTAG
polished    CTAGGGGATCGAAGAGGATTAGATACCCTCGTAGTCTTAGCTATAAACGATACCGACTAG
unpolished  CTAGGGGATCGAAGAGGATTAGATACCCTCGTAGTCTTAGCTATAAACGATACCGACTAG
            961......970.......980.......990.......1000......1010.......

U51151.1    GGGTTAGTAGAGGTCAAAGTGTCTTTATTAGTACCTTATGAGAAATCAAAGTCTTTGGGT
polished    GGGTTAGTAGAGGTCAAAGTGTCTTTATTAGTACCTTATGAGAAATCAAAGTCTTTGGGT
unpolished  GGGTTAGTAGAGGTCAAAGTGTCTTTATTAGTACCTTATGAGAAATCAAAGTCTTTGGGT
            1021.....1030......1040......1050......1060......1070.......

U51151.1    TCCGGGGGGAGTATGGTCGCAAGGCTGAAACTTAAAGGAATTGACGGAAGGGCACCACCA
polished    TCCGGGGGGAGTATGGTCGCAAGGCTGAAACTTAAAGGAATTGACGGAAGGGCACCACCA
unpolished  TCC--GGGGAGTATGGTCGCAAGGCTGAAACTTAAAGGAATTGACGGAAGGGCACCACCA
            1081.....1090......1100......1110......1120......1130.......

U51151.1    GGAGTGGAGCCTGCGGCTTAATTTGACTCAACACGGGGAAACTTACCAGGTCCAGACATA
polished    GGAGTGGAGCCTGCGGCTTAATTTGACTCAACACGGGGAAACTTACCAGGTCCAGACATA
unpolished  GGAGTGGAGCCTGCGGCTTAATTTGACTCAACAC-GGGAAACTTACCAGGTCCAGACATA
            1141.....1150......1160......1170......1180......1190.......

U51151.1    GGAAGGATTGACAGATTGATAGCTCTTTCTTGATTCTATGGGTGGTGGTGCATGGCCGTT
polished    GGAAGGATTGACAGATTGATAGCTCTTTCTTGATTCTATGGGTGGTGGTGCATGGCCGTT
unpolished  GGAAGGATTGACAGATTGATAGCTCTTTCTTGATTCTATGGGTGGTGGTGCATGGCCGTT
            1201.....1210......1220......1230......1240......1250.......

U51151.1    CTTAGTTGGTGGAGTGATTTGTCTGGCTAATTCCGATAACGAACGAGACCTCCGCCTTTA
polished    CTTAGTTGGTGGAGTGATTTGTCTGGCTAATTCCGATAACGAACGAGACCTCCGCCTTTA
unpolished  CTTAGTTGGTGGAGTGATTTGTCTGGCTAATTCCGATAACGAACGAGACCTCCGCCTTTA
            1261.....1270......1280......1290......1300......1310.......

U51151.1    ACTAGTGACGTGTATTGTGATATGCGTTGCTTCTTATAGGGACACTATATGTAAAATGTA
polished    ACTAGTGACGTGTATTGTGATATGCGTTGCTTCTTATAGGGACACTATATGTAAAATGTA
unpolished  ACTAGTGACGTGTATTGTGATATGCGTTGCTTCTTATAGGGACACTATATGTAAAATGTA
            1321.....1330......1340......1350......1360......1370.......

U51151.1    GGGAAGCTGGAGGCAATAACAGGTCTGTGATGCCCTTAGATGTTCTGGGCTGCACGCGCG
polished    GGGAAGCTGGAGGCAATAACAGGTCTGTGATGCCCTTAGATGTTCTGGGCTGCACGCGCG
unpolished  GGGAAGCTGGAGGCAATAACAGGTCTGTGATGCCCTTAGATGTTCTGGGCTGCACGCGCG
            1381.....1390......1400......1410......1420......1430.......

U51151.1    CGACACTGATCTATTCAACGAGTGGCTGGGTCGAGAGACTTGGCAAATCTTGTGAAAGTA
polished    CGACACTGATCTATTCAACGAGTGGCTGGGTCGAGAGACTTGGCAAATCTTGTGAAAGTA
unpolished  CGACACTGATCTATTCAACGAGTGGCTGGGTCGAGAGACTTGGCAAATCTTGTGAAAGTA
            1441.....1450......1460......1470......1480......1490.......

U51151.1    GATCGTGATGGGGATTGATGCTTGTAATTGTTCATCATGAACGAGGAATTCCTAGTAAAC
polished    GATCGTGATGGGGATTGATGCTTGTAATTGTTCATCATGAACGAGGAATTCCTAGTAAAC
unpolished  GATCGTGATGGGGATTGATGCTTGTAATTGTTCATCATGAACGAGGAATTCCTAGTAAAC
            1501.....1510......1520......1530......1540......1550.......

U51151.1    GCAAGTCATCAACTTGCATTGATTACGTCCCTGCCCTTTGTACACACCGCCCGTCGCACC
polished    GCAAGTCATCAACTTGCATTGATTACGTCCCTGCCCTTTGTACACACCGCCCGTCGCACC
unpolished  GCAAGTCATCAACTTGCATTGATTACGTCCCTGCCCTTTGTACACACCGCCCGTCGCACC
            1561.....1570......1580......1590......1600......1610.......

U51151.1    TACCGATTGAATGGTCCGGTGAACACTTTGGATTTGAGATTGGTTGTCTGTAAAAGGATG
polished    TACCGATTGAATGGTCCGGTGAACACTTTGGATTTGAGATTGGTTGTCTGTAAAAGGATG
unpolished  TACCGATTGAATGGTCCGGTGAACACTTTGGATTTGAGATTGGTTGTCTGT-AAAGGATG
            1621.....1630......1640......1650......1660......1670.......
```

```
U51151.1   GTTAATCTGGGAGAAGTCGTGTAAATCTTATCATTTAGAGGAAGGTGAAGTCGTAACAAG
polished   GTTAATCTGGGAGAAGTCGTGTAAATCTTATCATTTAGAGGAAGGTGAAGTCGTAACAAG
unpolished GTTAATCTGGGAGAAGTCGTGTAAATCTTATCATTTAGAGGAAGGTGAAGTCGTAACAAG
           1681.....1690......1700......1710......1720......1730.......

U51151.1   GTTTCCGTAGGTGAACCTGCGGAAGGATCA------------------------------
polished   GTTTCCGTAGGTGAACCTGCAGAAGGATCA------------------------------
unpolished GTTTCCGTAGGTGAACCTGCAGAAGGATCACCTGCAGGTGAAACCTTGTTTTGACTTCAC
           1741.....1750......1760......1770......1780......1790.......

U51151.1   ------------------------------------------------------------
polished   ------------------------------------------------------------
unpolished CTTCCTCTAAATGATAAGATTTACACGACTTCTCCCAGATTAACCATCCTTTACAGACAA
           1801.....1810......1820......1830......1840......1850.......

U51151.1   ------------------------------------------------------------
polished   ------------------------------------------------------------
unpolished CCAATCTCAAATCAAAGTGTTCACCGGACCATTCAATCGGCAGGTGCGACGAGCGGTGTG
           1861.....1870......1880......1890......1900......1910.......

U51151.1   --------------------------------------------------
polished   --------------------------------------------------
unpolished TACAAAGGGCAGGGACGTAATCAATGCAAGTTGATGACTTGCGTTTACTA
           1921.....1930......1940......1950......1960.......
```
